# Profiling Redox and Energy Coenzymes in Whole Blood, Tissue and Cells Using NMR Spectroscopy

**DOI:** 10.3390/metabo8020032

**Published:** 2018-05-14

**Authors:** G. A. Nagana Gowda

**Affiliations:** 1 Northwest Metabolomics Research Center, Department of Anesthesiology and Pain Medicine, University of Washington, Seattle, WA 98109, USA; ngowda@uw.edu; 2 Mitochondria and Metabolism Center, Department of Anesthesiology and Pain Medicine, University of Washington, Seattle, WA 98109, USA

**Keywords:** NMR, redox coenzymes, energy coenzymes, antioxidants, quantitation, blood, tissue, cells

## Abstract

Coenzymes of cellular redox reactions and cellular energy, as well as antioxidants mediate biochemical reactions fundamental to the functioning of all living cells. Conventional analysis methods lack the opportunity to evaluate these important redox and energy coenzymes and antioxidants in a single step. Major coenzymes include redox coenzymes: NAD^+^ (oxidized nicotinamide adenine dinucleotide), NADH (reduced nicotinamide adenine dinucleotide), NADP^+^ (oxidized nicotinamide adenine dinucleotide phosphate) and NADPH (reduced nicotinamide adenine dinucleotide phosphate); energy coenzymes: ATP (adenosine triphosphate), ADP (adenosine diphosphate) and AMP (adenosine monophosphate); and antioxidants: GSSG (oxidized glutathione) and GSH (reduced glutathione). We show here that a simple ^1^H NMR experiment can measure these coenzymes and antioxidants in tissue and whole blood apart from a vast pool of other metabolites. In addition, focused on the goal of identification of coenzymes in subcellular fractions, we demonstrate analysis of coenzymes in the cytoplasm using breast cancer cells. Owing to their unstable nature, or low concentrations, most of the coenzymes either evade detection or lose their integrity when established sample preparation and analysis methods are used. To overcome this challenge, here we describe the development of new methods to detect these molecules without affecting the integrity of other metabolites. We used an array of 1D and 2D NMR methods, chemical shift databases, pH measurements and spiking with authentic compounds to establish the identity of peaks for the coenzymes and antioxidants in NMR spectra. Interestingly, while none of the coenzymes and antioxidants were detected in plasma, they were abundant in whole blood. Considering that the coenzymes and antioxidants represent a sensitive measure of human health and risk for numerous diseases, the presented NMR methods to measure them in one step potentially open new opportunities in the metabolomics field.

## 1. Introduction

The field of metabolomics deals with the simultaneous analysis of a large pool of small molecules that are the downstream products of the functions of genes, transcripts and enzymes. The knowledge of concentration of the small molecules, known as metabolites, enables gaining insights into their role in health and pathogenesis of numerous human diseases. Biological specimens including a variety of biofluids, tissue and cells are employed for metabolomics investigations. Pivotal to the metabolomics studies are the analytical methods that enable reliable and reproducible measurement of metabolites. Two analytical platforms that are widely used in the metabolomics field, currently, are mass spectrometry (MS) and NMR spectroscopy. The two platforms offer complementary capabilities. MS is highly sensitive and it enables measurement of several hundreds to thousands of metabolites in a single measurement routinely. NMR, on the other hand, is highly quantitative and reproducible and enables identification of unknown compounds in complex biological mixtures by using a combination of an array of 1D/2D techniques within the NMR platform. Owing to the increasingly realized complexity of biological specimens, interest to explore the combined strength of the two analytical platforms is on the rise.

One major aspect of the metabolomics field is the development of new methods that enable identification and quantitation of metabolites, which are difficult to detect. Among important metabolites fundamental to the functioning of all living cells are the coenzymes of cellular redox reactions, coenzymes of cellular energy and antioxidants. Specifically, these classes of compounds include redox coenzymes: NAD^+^ (oxidized nicotinamide adenine dinucleotide), NADH (reduced nicotinamide adenine dinucleotide), NADP^+^ (oxidized nicotinamide adenine dinucleotide phosphate) and NADPH (reduced nicotinamide adenine dinucleotide phosphate); energy coenzymes: ATP (adenosine triphosphate), ADP (adenosine diphosphate) and AMP (adenosine monophosphate); and antioxidants: GSSG (oxidized glutathione) and GSH (reduced glutathione). Despite numerous advances in the metabolomics field, no simple method existed for their simultaneous analysis. Conventionally, the coenzymes are measured individually, which has the high risk of measurement errors outweighing biological changes. Efforts have been made to analyze them in one step using MS [1,2]. However, major challenges for MS in reliable quantification of these coenzymes are ion suppression and peak interference. In particular, NAD^+^ and NADP^+^ signals overlap in the first quadrupole (Q1) with NADH and NADPH, respectively, and vice versa due to unit mass differences. In addition, for the energy coenzymes, in-source fragmentation of ATP to ADP and AMP, and ADP to AMP during MS analysis hinders their reliable measurement [2]. 

In contrast, NMR spectroscopy offers numerous benefits including the ability to reliably identify and simultaneously quantify many compounds in complex biological mixtures with high reproducibility and quantitative accuracy [3,4,5,6,7,8,9]. NMR analysis is non-invasive, so the sample is retained after the data acquisition and can be used for any other further analysis. We have recently demonstrated the quantitation of metabolites down to sub-micromolar levels [10,11]. However, the coenzymes were not amenable for analysis using NMR owing to numerous challenges including the low concentration of some of the coenzymes and similarity in molecular structures, which affected reliable peak identification. In order to analyze the coenzymes, it was necessary to establish their fingerprint and, in particular, isolated peaks that can be used for quantifying them on a routine basis. Another major challenge unconnected with measurement methods is the instability of several coenzymes including the NADH, NADPH and ATP due to their sensitivity to sample harvesting and metabolite extraction protocols. Nevertheless, in separate studies using different types of tissues, cells and whole blood, we have overcome these bottlenecks and provided simple NMR methods to quantify many coenzymes and antioxidants, simultaneously, in addition to a vast pool of other metabolites [12,13]. Studies were also focused on detecting coenzymes in subcellular levels. In the following sections, we describe these methods with an emphasis on the analysis of redox coenzymes, energy coenzymes and antioxidants.

## 2. Coenzymes Analysis in Tissue

Coenzymes analysis in tissue provides opportunity to visualize mitochondrial activity and function. For cellular energy, ATP synthesis is a major function of the mitochondria. To achieve ATP synthesis in the mitochondrial respiration chain, the coenzyme NADH oxidizes to NAD^+^ through the electron transport chain and, simultaneously, the ADP is converted to ATP through oxidative phosphorylation. In the human body, the amount of ATP produced and consumed in 24 h is approximately equal to the weight of the human body itself. This signifies the need and magnitude of ATP production. It also highlights the importance of the coenzymes in mitochondrial function and the need to be able to visualize their levels simultaneously using a simple method on a routine basis.

**Figure 1:**
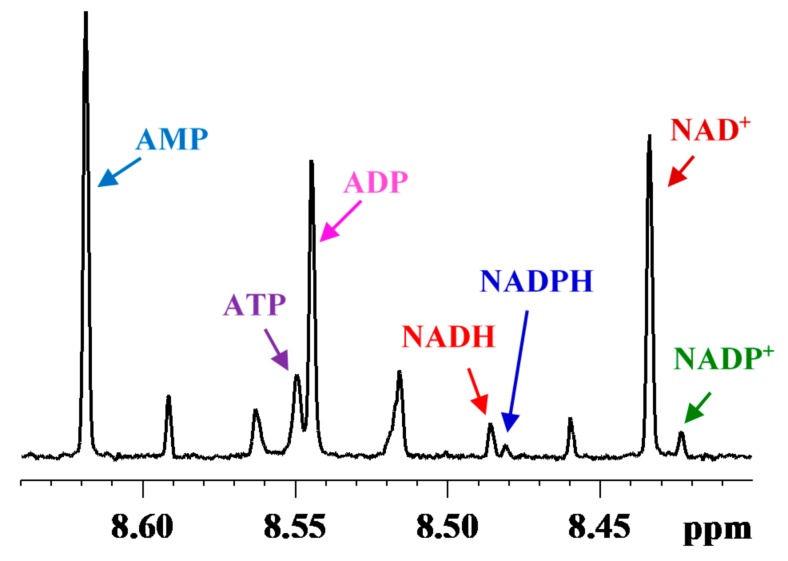
Portion of a typical 800 MHz ^1^H NMR spectrum of a mouse liver tissue, obtained before optimizing the tissue harvesting and extraction method showing characteristic peaks for coenzymes: NAD^+^ (oxidized nicotinamide adenine dinucleotide), NADH (reduced nicotinamide adenine dinucleotide), NADP^+^ (oxidized nicotinamide adenine dinucleotide phosphate), NADPH (reduced nicotinamide adenine dinucleotide phosphate); ATP (adenosine triphosphate), ADP (adenosine diphosphate) and AMP (adenosine monophosphate).

A number of studies in our laboratory were focused on metabolite identification in complex biological mixtures and their quantitation using NMR [10,11]. The strategies used in these studies formed the basis for the identification and quantitation of the coenzymes using NMR. Various types of mouse tissue including heart, liver, kidney, skeletal muscle and brain tissue were employed for the development of coenzymes analysis method. Optimization of tissue harvesting and metabolite extraction protocols were critical for the analysis of the coenzymes. This is due to the fact that many coenzymes are unstable and depending on the protocol used coenzymes such as NADH and NADPH were oxidized wholly or partly to NAD^+^ and NADP^+^, respectively; in addition, most of the ATP was converted to ADP and AMP as shown in Figure 1. 

**Figure 2:**
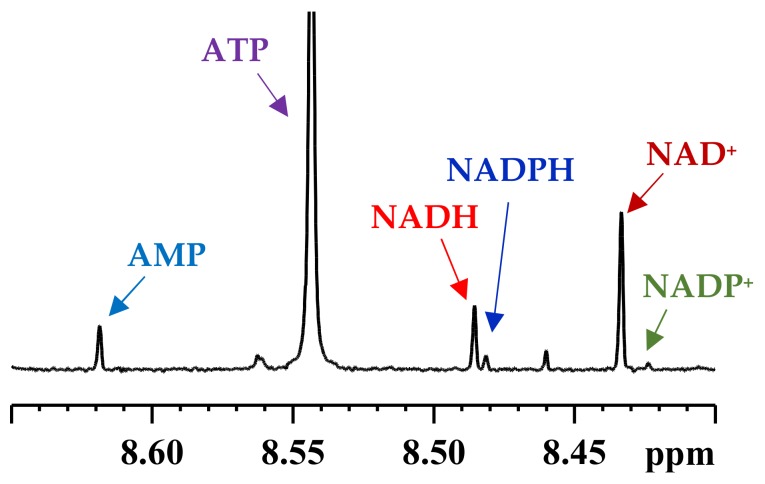
Portion of a typical 800 MHz ^1^H NMR spectrum of a mouse heart tissue showing characteristic peaks for the coenzymes: NAD^+^ (nicotinamide adenine dinucleotide, oxidized), NADH (nicotinamide adenine dinucleotide, reduced), NADP^+^ (nicotinamide adenine dinucleotide phosphate, oxidized), NADPH (nicotinamide adenine dinucleotide phosphate, reduced); ATP (adenosine triphosphate), ADP (adenosine diphosphate) and AMP (adenosine monophosphate).

Several combination of tissue harvesting and extraction methods were initially used to optimize the methods. Important tissue harvesting protocols for the mouse heart included: (1) In vivo freeze clamping with no perfusion of the heart; (2) Langendorff isolated heart perfusion [14] followed by in vivo freeze clamping; (3) washing isolated hearts with a glucose (10 mM) and pyruvate (0.5 mM) solution followed by freeze clamping; and (4) washing isolated hearts with phosphate buffer saline solution followed by freeze clamping. Extraction protocols were investigated using different solvent combinations including a mixture of methanol and water, a mixture of methanol and chloroform, and perchloric acid. The performance of each harvesting and extraction method was gauged by monitoring ^1^H NMR spectra. It was found that immediately washing the harvested tissue with a solution containing a mixture of glucose (10 mM) and pyruvate (0.5 mM) and freeze clamping followed by extraction using a mixture of methanol and chloroform (1:1 *v*/*v*) and then a mixture of distilled water and chloroform (1:1 *v*/*v*) provided the best results for detecting all the coenzymes [12]. A large intact ATP signal in the spectrum is an indicator of the integrity of the tissue harvesting and extraction method as shown in Figure 2. 

**Figure 3:**
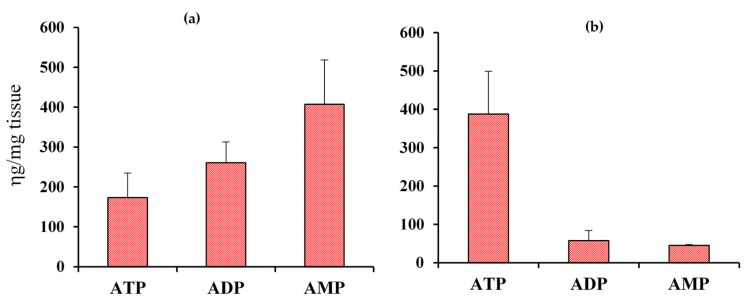
Absolute concentrations of ATP, ADP, AMP in heart tissue of wild type mice obtained using the new ^1^H NMR spectroscopy method (**a**) before optimizing the harvesting and extraction method (n = 4); and (**b**) after optimizing the harvesting and extraction method (n = 6).

As an example, Figure 3 compares the energy coenzymes, ATP, ADP and AMP, obtained from mouse heart tissue samples before and after optimization of the harvesting and extraction method. It is particularly striking that the total pool of energy coenzymes is significantly higher when the protocols were not optimized, which potentially indicates that active metabolism, in the absence of proper quenching of tissue, causes accumulation of AMP/ADP/ATP pool.

**Figure 4:**
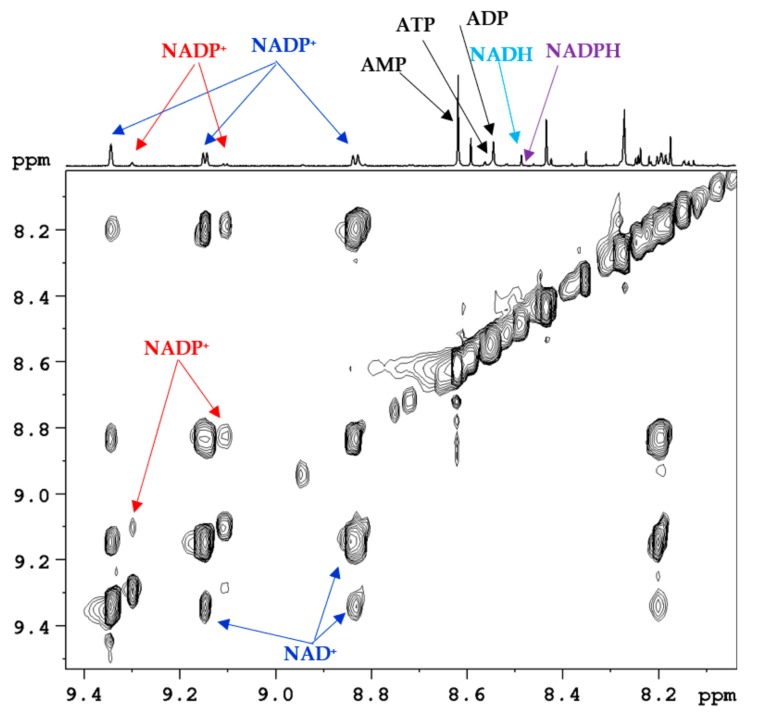
Portion of a typical 800 MHz ^1^H-^1^H 2D TOCSY NMR spectrum along with a trace of the corresponding 1D spectrum obtained using mouse kidney tissue with highlighting some of the peaks of coenzymes: NAD^+^ (oxidized nicotinamide adenine dinucleotide), NADP^+^ (oxidized nicotinamide adenine dinucleotide phosphate). NADH (reduced nicotinamide adenine dinucleotide), NADPH (reduced nicotinamide adenine dinucleotide phosphate), ATP (adenosine triphosphate), ADP (adenosine diphosphate) and AMP (adenosine monophosphate).

Comprehensive analysis combining ^1^H 1D NMR, ^1^H-^1^H 2D DQF-COSY (double quantum filtered correlated spectroscopy) and ^1^H-^1^H 2D TOCSY (total correlated spectroscopy) experiments, chemical shift databases, and spiking with authentic compounds resulted in the unambiguous identification of all the coenzymes. Portion of a typical ^1^H-^1^H 2D TOCSY NMR spectrum along with the 1D trace obtained using a mouse kidney tissue is shown in Figure 4 with highlighting of some of the NMR peaks. In view of the very similar structures for many coenzymes, for better comparison, the chemical shift library used for initial peak assignment was developed using solutions of standard compounds, whose concentrations were kept very close to their physiological levels in tissue.

Tissue extraction protocol and coenzymes analysis using NMR method were evaluated based on reproducibility and recovery experiments. To demonstrate the reproducibility, NMR spectra of many tissue samples (n = 6) obtained using identical protocol were tested, whereas for the recovery test, tissue samples spiked with standard compounds were evaluated. 

### 2.1. Coenzyme Levels in Mice with Cardiac Specific Knockout of the Mitochondrial Complex I Ndufs4 Gene (cKO)

The newly developed tissue harvesting and extraction protocols were then applied for the coenzymes analysis in mouse heart tissue using the NMR method. Specifically, to demonstrate the performance of the new method, we measured cardiac NADH and NAD^+^ ratios as well as their pool sizes in heart tissue from mouse models with a cardiac specific knockout of the mitochondrial Complex I Ndufs4 gene (cKO). The results were compared with those for wild-type (WT) mouse obtained under identical conditions.

**Figure 5:**
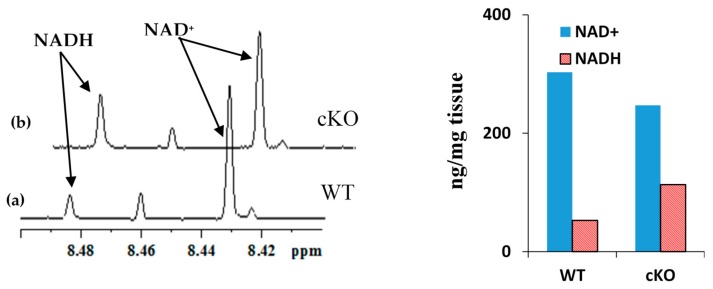
(**a**) Portion of typical 800 MHz NMR spectra of mouse heart tissue extracts highlighting characteristic NAD^+^ and NADH peaks in (**a**) WT and (**b**) cKO mice obtained under identical conditions; the bar chart on the right highlights increased NADH/NAD^+^ ratio in cKO mouse without altering the pool size as compared to WT mouse. In WT mouse, the NAD(H) pool size was 356 ng/mg tissue and the NADH/NAD^+^ ratio was 0.17; in cKO mouse, the NAD(H) pool size was 360 ng/mg tissue and the NADH/NAD^+^ ratio was 0.46.

Figure 5 shows portions of NMR spectra highlighting characteristic peaks for the NADH and NAD^+^ in a cKO and WT mouse along with their concentrations. In WT mouse, the NAD(H) pool size was 356 ng/mg tissue and the NADH/NAD^+^ ratio was 0.17, whereas in cKO mouse, the NAD(H) pool size was 360 ng/mg tissue and the NADH/NAD^+^ ratio was 0.46. In cKO mouse the NADH concentration increased relative to WT mouse, which results in increased NADH/NAD^+^ ratio relative to WT. This is due to cKO gene results in inhibition of mitochondrial complex I activity and accumulation of NADH in the mitochondria [15].

### 2.2. Coenzyme Levels in Transgenic Mice with Overexpression of Nicotinamide Phosphoribosyltransferase (cNAMPT)

**Figure 6:**
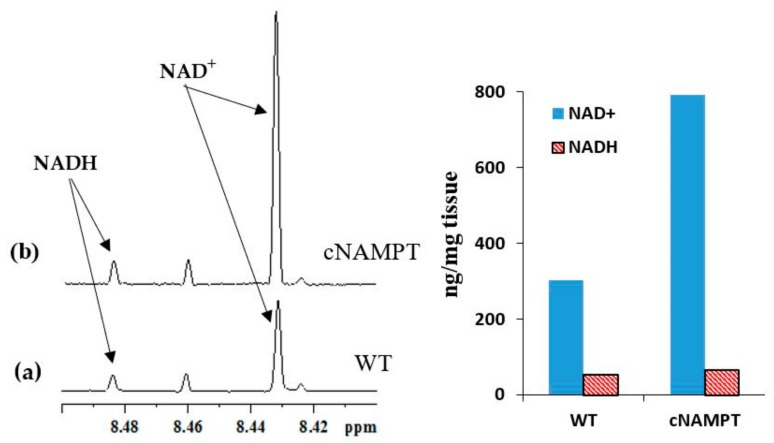
(**a**) Portion of typical 800 MHz NMR spectra of heart tissue extracts highlighting the NAD^+^ and NADH peaks in WT and (**b**) cNAMPT mice obtained under identical experimental conditions; The bar chart on the right highlights the increased NAD(H) pool size in cNAMPT mice without altering NADH/NAD^+^ ratio relative to WT mice, appreciably. In WT mouse, the NAD(H) pool size was 356 ng/mg tissue and the NADH/NAD^+^ ratio was 0.17, whereas in cNAMPT mouse, the NAD(H) pool size was 858 ng/mg tissue and the NADH/NAD^+^ ratio was 0.08.

The analysis method was also tested for heart tissue from transgenic mouse models with cardiac specific overexpression of nicotinamide phosphoribosyltransferase (cNAMPT) [16]. Overexpression of cNAMPT results in elevation of the NAD^+^ levels and the NAD(H) pool size. Figure 6 compares the NADH and NAD^+^ peaks between the transgenic mice with overexpression of cNAMPT and WT mice. In cNAMPT mouse, the NAD(H) pool size was 858 ng/mg tissue and the NADH/NAD^+^ ratio was 0.08 as compared to 356 ng/mg tissue and 0.17, respectively, in WT mouse. As anticipated the NAD(H) pool size was increased significantly in cNAMPT mouse relative to WT mouse. However, although, the NADH/NAD^+^ ratio in cNAMPT mouse was anticipated to be identical to that observed in WT mouse, the experimentally derived value was marginally lower. Such deviation may be attributed to the biological variation between the two genotypes. 

### 2.3. Coenzyme Levels in Mice with Cardiac Specific Knockout of the Mitochondrial Complex I Ndufs4 Gene (cKO) and Cardiac Specific Overexpression of Nicotinamide Phosphoribosyltransferase (cNAMPT)

**Figure 7:**
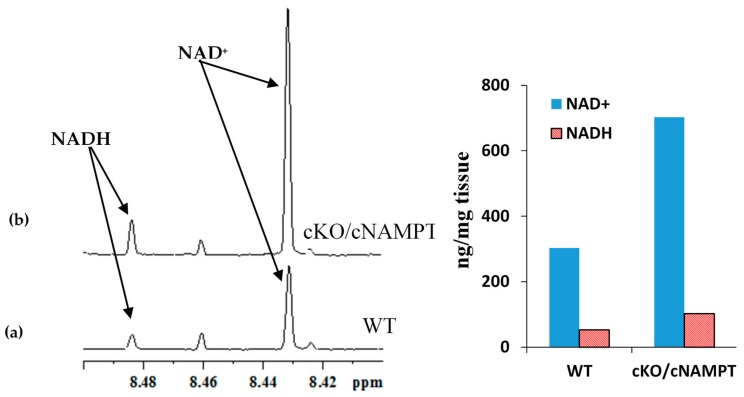
(**a**) Portions of typical 800 MHz NMR spectra of mouse heart tissue extracts highlighting the NAD^+^ and NADH peaks in WT and (**b**) cKO/cNAMPT mice obtained under identical experimental conditions; The bar chart shown on the right highlights the changes in the NAD^+^/NADH pool size and the NADH/NAD^+^ ratio in cKO/cNAMPT relative to WT mouse. In WT mouse, the NAD(H) pool size was 356 ng/mg tissue and the NADH/NAD^+^ ratio was 0.17; in cKO/cNAMPT mouse, the NAD(H) pool size was 805 ng/mg tissue and the NADH/NAD^+^ ratio was 0.14.

We have also tested coenzyme levels in mice that possessed both cardiac specific knockout of the mitochondrial complex I *Ndufs4* gene (cKO) and the transgene for cardiac specific overexpression of nicotinamide phosphoribosyltransferase (cNAMPT). Such mice are known to exhibit increased NADH/NAD^+^ ratio relative to WT mice due to the inhibition of Complex I activity arising from Ndufs4 gene knockout; in addition, they exhibit increased NAD(H) pool size relative to WT mice due to the transgene that overexpresses nicotinamide phosphoribosyltransferase. Results obtained from the analysis of the coenzymes using NMR are shown in Figure 7. In cKO/cNAMPT mouse, the NAD(H) pool size was 805 ng/mg tissue and NADH/NAD^+^ ratio was 0.14. As anticipated the NAD(H) pool size in cKO/cNAMPT mouse was increased significantly (805 ng/mg tissue) relative to WT mouse (356 ng/mg tissue). Further, as anticipated, the NADH/NAD^+^ ratio in cKO/cNAMPT (0.14) was higher by a factor of nearly 2 when compared cNAMPT mouse (0.08) (Figure 6 and Figure 7).

The new NMR method enables quantification of major coenzymes and antioxidants in a single experiment, apart from a vast pool of other metabolites. This is significant considering that conventional methods such as enzymatic assays increase the likelihood of errors since each coenzyme needs to be analyzed separately in this method. It may, however, be noted that the NMR method measures both free and bound forms of the coenzymes since the use of organic solvents results in extraction of both forms. Hence, the measured coenzymes concentrations by this method represent the sum of free and bound forms.

## 3. Coenzymes in Human Whole Blood

We recently extended the method developed for coenzymes analysis in tissue extracts to their analysis in blood. Human blood is widely used in the metabolomics field for investigations of virtually all human diseases. However, owing to the need for simple sample processing, in virtually all blood-based metabolomics studies, metabolites are generally measured in blood plasma or serum. The cellular components of the blood including red blood cells (RBC), white blood cells and platelets are thus discarded during sample processing. In blood, more than 99% of the cells are RBC and RBC account for nearly 50% of total blood volume. A major drawback of metabolomics approach that uses serum/plasma analysis is the lack of the ability to analyze important coenzymes of redox reactions, coenzymes of energy and antioxidants. 

**Figure 8:**
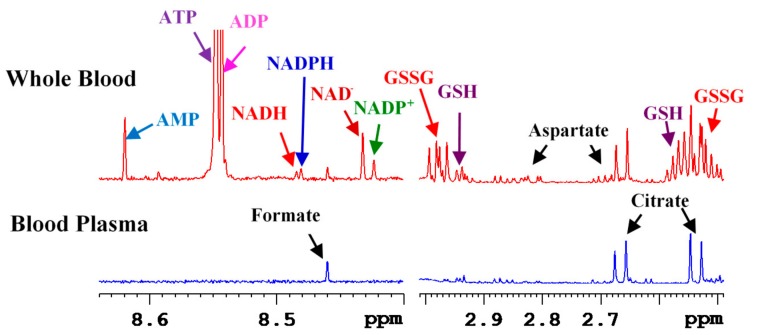
Portions of typical 800 MHz ^1^H NMR spectra of extracts of whole blood and blood plasma from the same individual. The peak identities for redox and energy coenzymes and antioxidants in the extract of whole blood are indicated. NAD^+^: nicotinamide adenine dinucleotide, oxidized; NADH: nicotinamide adenine dinucleotide, reduced; NADP^+^: nicotinamide adenine dinucleotide phosphate, oxidized; NADPH: nicotinamide adenine dinucleotide phosphate, oxidized; ATP: adenosine triphosphate; ADP: adenosine diphosphate; AMP: adenosine monophosphate; GSH: glutathione, reduced; and GSSG: glutathione, oxidized. Note that none of these compounds were detected in blood plasma.

We hypothesized that whole blood provides access to wider pool of metabolites including coenzymes with no additional efforts. Thus, we developed an NMR method for whole blood metabolites analysis. The new method combines the protocols developed for tissue analysis [12] as well as the protocols developed for serum/plasma analysis [10]; while the tissue analysis protocol enabled coenzymes analysis in one step, serum/plasma analysis protocol provided an expanded pool of quantifiable metabolites (>70). Briefly, for extraction of whole blood metabolites, 200 to 400 μL whole blood was mixed with methanol/chloroform in a 1:2:2 (*v*/*v*/*v*) ratio, vortexed, sonicated for 2 min and the mixture was kept aside for 20 min at −20 °C. The mixture was then centrifuged at 13,400 rcf for 30 min to pellet insoluble macromolecular components including the proteins and cell debris. The supernatant aqueous layer was separated, dried using a vacuum concentrator and mixed with 600 μL buffer containing 25 μM TSP (sodium salt of 3-(trimethylsilyl)propionic acid-2,2,3,3-d_4_) for NMR analysis. Identical procedure was used for the extraction of metabolites in blood plasma. Peak identities for the coenzymes, antioxidants and other metabolites in blood NMR spectra were established based on 1D and 2D NMR methods, chemical shift databases, pH measurements and spiking with authentic compounds. This resulted in the identification of virtually every peak in the 1D NMR spectrum of whole blood extracts. Among others, important metabolites identified in whole blood extracts were NAD^+^, NADH, NADP^+^, NADPH, ATP, ADP, AMP, GSH and GSSG. A comparison of whole blood and plasma NMR spectra revealed that the redox coenzymes, energy coenzymes and antioxidants were detected only in whole blood [13] (Figure 8).

The new approach to analyze whole blood metabolites is an important alternative to serum/plasma metabolomics and shows that redox and energy coenzymes and antioxidants can be analyzed simultaneously along with the nearly 70 metabolites, which were shown to be quantitated in serum/plasma, previously [10]. As the coenzymes were not detected in blood plasma and RBC are the major components of blood cells (>99%), virtually all the detected coenzymes come from the RBC.

An important aspect of whole blood analysis is that it provides metabolite levels in both blood plasma and RBC in a single measurement with virtually the same effort as used in the traditionally used serum or plasma analysis. Hence, whole blood metabolomics offers an added opportunity to gain insights into the important coenzymes and antioxidants. It is important to note that the coenzymes and antioxidants represent a sensitive measure of the cellular functions in human health and diseases, and the levels for all them can now be measured in blood, which is a minimally invasive approach. This new capability is significant in that there was no NMR or MS method that can enable analysis of major coenzymes and antioxidants in whole blood. The new NMR method is anticipated to pave new ways for blood metabolomics [13].

## 4. Subcellular Coenzymes

Global analysis of cells or tissue lacks insights into the subcellular levels and metabolism, which is a major bottleneck considering the increasingly realized roles of subcellular components in the pathogenesis of human diseases. The coenzymes, NAD^+^, NADH, NADP^+^ and NADPH mediate biochemical reactions fundamental to the functioning of all living cells. They undergo reversible oxidation and reduction, and the ratio of the concentrations of reduced and oxidized forms of coenzymes reflects important cellular functions including the overall redox status, regulation of ion channels, cell signaling, and cell survival or death [17,18,19,20]. The redox balance is also an important indicator of numerous pathological conditions including heart disease, diabetes and cancer. In particular, in the investigation of cancer, interest in targeting the NAD metabolism as a new therapeutic approach for cancer treatment is increasing owing to the close association of the NAD metabolome with cancer cell biology [21,22,23]. The NAD^+^/NADH ratio is closely associated with the function of mitochondrial Complex I and oxidative phosphorylation. The evidence that the NAD^+^/NADH balance is altered in cancer provides new avenues for cancer biomarkers detection as well as targets for anti-cancer therapies [24]. However, a bottleneck is the lack of understanding of the NAD metabolome specifically at the subcellular level, which in turn is due to the lack of a reliable analytical method. Our ability to evaluate coenzymes at the subcellular levels will not only enable identification of cancer biomarkers, but also opens avenues for the identification of potential enzyme targets for drug discovery.

### Isolation of Mitochondria and Cytoplasm and Extraction of Metabolites

We used the strategy developed for the analysis of coenzymes in tissue and blood as described above to analyze subcellular coenzymes. We utilized the non-cancerous breast epithelial cell lines (MCF10A) and two breast cancer cell lines (MCF7 and MDA-MB231) for isolation of subcellular components such as mitochondria and the cytoplasm. Cells were washed with cold PBS, pelleted by centrifugation, then resuspended in STM buffer comprised of sucrose (250 mM), Tris–HCl (50 mM) pH 7.4, MgCl_2_ (5 mM), protease and phosphatase inhibitor cocktails followed by homogenization on ice using a Teflon pestle. The homogenate was transferred into a centrifuge tube and maintained on ice for 30 min, vortexed and then centrifuged (800× *g* for 15 min). The pellet was separated and the supernatant was used to isolate mitochondrial and the cytosolic fractions by centrifugation at 800× *g* for 10 min. The resulted supernatant was then centrifuged at 11,000× *g* for 10 min to pellet the mitochondria, leaving the cytosolic fraction in the supernatant.

**Figure 9:**
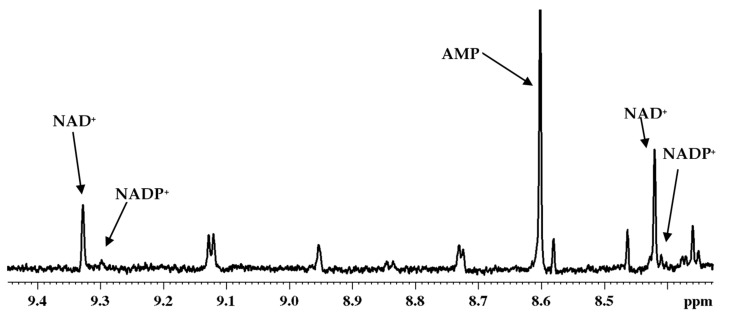
Portion of typical 800 MHz ^1^H NMR spectrum of the cytoplasm fraction isolated from breast cancer cells (MCF7). The identification of nicotinamide adenine dinucleotide, oxidized (NAD^+^); nicotinamide adenine dinucleotide phosphate, oxidized (NADP^+^) and adenosine monophosphate (AMP) are marked.

We have shown previously that the coenzymes are extremely labile and they can evade detection wholly or partly depending on the procedure used for extraction. In view of this, we followed the extraction protocol optimized for analysis of tissue and whole blood. In particular, we have shown that methanol and chloroform mixture arrests enzyme activity and provides more reliable results. We thus used this solvent mixture for extraction of the subcellular coenzymes. Figure 9 shows a portion of a typical ^1^H NMR spectrum highlighting the coenzymes detected in cytoplasm of breast cancer cells (MCF7).

The coenzyme peaks for the mitochondrial fractions, however, were very weak and hence a further optimization of the method is needed to detect them with high sensitivity. Identification of coenzymes in subcellular fractions is critical in view of their roles in human diseases, cancer in particular. Despite the immense need, there is a lack of studies focused on identifying subcellular coenzymes. Hence, our ability to measure them reliably offers new avenues for understanding their role in diseases such as cancer.

## 5. Conclusions

In this article, we describe methods developed recently that enable one–step analysis of redox coenzymes, energy coenzymes and antioxidants using ^1^H NMR spectroscopy in tissue, blood and cells. These methods have overcome numerous challenges including the instability of many coenzymes. Considering the critical role that the coenzymes play in various cellular functions and the numerous challenges associated with the conventional methods, the NMR methods presented in this study represent a new avenue to visualize coenzymes and antioxidants in various biological mixtures. We also demonstrate the sensitivity of redox coenzymes to different mouse genotypes as examples of the utility of the developed methods. Apart from coenzymes and antioxidants, whole blood metabolomics enables access to a vast pool of other metabolites and also avoids the confounding effect of hemolysis encountered in the conventional serum/plasma metabolomics. Importantly, the ability to measure the coenzymes using whole blood opens a new avenue for monitoring their levels on a routine basis using minimally invasive method.
